# Influence of a Combination of Glycerol Polyethylene Glycol Ricinoleate and Bi-Distilled Oleic Acid in Powder Form on Growth Performance, Nutrient Digestibility, Excreta Nitrogen and Liver Fatty Acid Profile of Broilers Fed Reduced-Energy Diets

**DOI:** 10.3390/ani15060827

**Published:** 2025-03-13

**Authors:** Luca Marchetti, Raffaella Rebucci, Caterina Piantoni, Paola Antonia Corsetto, Angela Maria Rizzo, Haijun Zhang, Xianren Jiang, Valentino Bontempo

**Affiliations:** 1 Department of Veterinary and Animal Science (DIVAS), Università degli Studi di Milano, Via dell’Università 6, 29600 Lodi, Italy; raffaella.rebucci@unimi.it (R.R.); caterina.piantoni@unimi.it (C.P.); valentino.bontempo@unimi.it (V.B.); 2 Department of Pharmacological and Biomolecular Sciences “Rodolfo Paoletti” (DiSFeB), Università degli Studi di Milano, Via Trentacoste 2, 20134 Milan, Italy; paola.corsetto@unimi.it (P.A.C.); angelamaria.rizzo@unimi.it (A.M.R.); 3 Key Laboratory of Feed Biotechnology of the Ministry of Agriculture, Institute of Feed Research, Chinese Academy of Agricultural Sciences, Beijing 100081, China; zhanghaijun@caas.cn (H.Z.); jiangxianren@caas.cn (X.J.)

**Keywords:** fats, emulsion, nutrition, digestibility, soybean oil, body weight, oleic acid, linoleic acid

## Abstract

The present study aimed to evaluate the effects of an emulsifier in powder form composed of glycerol polyethylene glycol ricinoleate and bi-distilled oleic acid on the growth performance, nutrient digestibility, excreta nitrogen and liver fatty acid profile of broilers fed reduced-energy diets. A total of 720 male ROSS 308 chicks were divided into four homogeneous groups (9 pens/20 chicks per group): positive control (PC), negative control (NC, −70 kcal/kg of AME), EMUL1 and EMUL2 fed an NC diet + 250 and 500 mg/kg of emulsifier. Body weight, average daily gain, average daily feed intake and feed conversion ratio (FCR) were evaluated. Excreta were collected at 24 and 42 d directly from polyethylene trays and pooled to determine the apparent total tract digestibility of dry matter, crude protein, ether extract and gross energy. The apparent metabolizable energy (AME, AMEn) and nitrogen ammonia in excreta were determined. Performances were improved in EMUL1 and EMUL2 during the trial (*p* < 0.05). The digestibility of nutrients in EMUL1 and EMUL2 was increased in comparison to NC and PC (*p* < 0.05). Excreta nitrogen ammonia was reduced by EMUL2 at 42 d (*p* < 0.05). Glycerol polyethylene glycol ricinoleate and bi-distilled oleic acid in powder form modulated liver fatty acid profiles, enhancing oleic acid content in EMUL1 chickens (*p* < 0.05), while γ-linolenic acid and arachidonic acid were enhanced in EMUL2 (*p* < 0.05). Based on the findings, it is recommended to administer solidified glycerol polyethylene glycol ricinoleate and bi-distilled oleic acid in diets with reduced energy content for appreciable advantages in terms of growth through higher nutrient digestibility and better hepatic fatty acid composition.

## Simple Summary

Emulsifiers may contribute to reducing the inclusion of complex energy sources, such as soybean oil, in broiler diets by increasing nutrient digestibility. The present study evaluated the effects of a combination of glycerol polyethylene glycol ricinoleate and bi-distilled oleic acid in powder form when supplemented in broiler diets with reduced energy content. The results highlight the possibility to improve growth performance of broilers through better nutrient digestibility while reducing soybean oil dietary inclusion. Therefore, the tested emulsifier may contribute in a more sustainable way to broiler nutrition.

## 1. Introduction

Emulsifiers play a crucial role in poultry nutrition by enhancing fat digestion and absorption, leading to improved feed efficiency and growth performance [1,2,3]. These compounds help in emulsifying dietary fats, increasing their surface area for better digestive enzyme action and ensuring efficient utilization of energy from feed ingredients [4]. The inclusion of emulsifiers in poultry diets has been linked to better nutrients absorption, improved feed conversion ratios and optimized production efficiency. Additionally, emulsifiers can support gut health by modulating lipid metabolism and reducing the risk of fat-related digestive disorders in chickens [1,2,3,4,5].

Interestingly, it was previously shown and discussed how feed additives such as emulsifiers can cope with dietary energy decreases in broilers [5,6]. Reducing dietary energy while increasing nutrient digestibility may represent an ideal strategy to improve feed ingredient utilization, with positive reflexes on chicken’s gut health and the growth of birds [6]. Nevertheless, various kinds of additives are being used in livestock production not only to enhance productivity but to maintain sustainability and to reduce environmental impact [7,8,9]. However, there is a growing interest in optimizing fats’ supplementation with various kinds of additives to ameliorate the dietary energy utilization for high-performing modern breeds of broilers [1,2,3,4]. Fats and oils are the most calorie-dense nutrients among all nutrients that are provided in poultry feed [10].

Unlike other macronutrients, the digestion and absorption of fats involve diverse physicochemical pathways in which fat droplet breakdown, lipolysis and micelle formation are crucial [11,12]. Moreover, the inclusion of fats in broiler diets can enhance the assimilation and utilization of fat-soluble vitamins and other nutrients, contributing to the improvement of gut health conditions and growth performances [11,12]. Fats are water-insoluble, and to become accessible for enzymatic breakdown by lipase they require emulsification, which is influenced by factors like the length of the fatty acid chains, the esterification of fatty acids with triglycerides and the degree of fat saturation [12,13]. Furthermore, in young birds there is a lack of bile salts and lipase production, which causes a reduction in fat digestion [1]. However, while the hydrolysis of triacylglycerols is followed by the passive absorption in the intestinal lumen of a part of the resulting products, long-chain saturated fatty acids and diacylglycerols need endogenous emulsification [10,11,12]. Nevertheless, existing data suggest that the capacity for fat digestion and absorption in chickens is limited immediately after hatching and that they gradually improve [1,2,3,4].

It was previously highlighted how exogenous emulsifiers supplemented in poultry diets have the potential to enhance growth performance by improving fat utilization [1,2,3,4]. Emulsifiers are surfactants that promote the formation of stable emulsions between two substances that are not normally miscible [14,15]. Briefly, acting as polar amphipathic molecules with both hydrophilic and hydrophobic properties, emulsifiers can enhance fat utilization. Therefore, the administration of emulsifiers can facilitate the digestion of fats during the different growth stages of chickens [16,17]. Furthermore, previous research has revealed that the inclusion of emulsifiers, such as lysophopholipids and lysolecithins, effectively mitigated the performance loss when dietary metabolizable energy (ME) was reduced in comparison to optimal levels [18,19]. Nonetheless, glycerol polyethylene glycol ricinoleate is known as one of the most used emulsifiers in food and feed production chains due to its nutritional and technological properties [3].

Moreover, the stabilization of viscous-liquid emulsifiers on silica through nebulization may bring appreciable technological advantages. The interaction among surfactants and solid carriers may influence their emulsification properties [20]. In addition, polyethylene glycol monoalkyl ethers can form surface micelles or bilayer aggregates when absorbed on hydrophilic surfaces such as silica [20].

Silica-based solid carriers can modulate the emulsion activity and stability of glycerol polyethylene glycol ricinoleate [20,21]. Moreover, it is expected that solidified glycerol polyethylene glycol ricinoleate and bi-distilled oleic acid may further reduce the surface tension of water, increasing its penetration and distribution in pressed feeds during conditioning and pelleting processes in feed mills [22]. Therefore, it is reasonable to consider that solidified emulsions could be more easily miscible before pelleting processes with other feed ingredients, leading to further technological advantages due to homogenous distribution after mixing, better moisture modulation, lower energy expenses and easier transport and application [23]. 

Soybean oil represents one of the most common sources of lipids used in feeds [24]. Nonetheless, soybean oil production involves notable environmental impact within the feed production chain, even considering alternative methods of production [25,26]. Therefore, reducing the soybean oil dietary inclusion while optimizing lipid digestion and energy utilization may represent an ideal strategy to promote a more sustainable approach to broiler nutrition.

Considering the overall background, after evaluating liquid forms of glycerol polyethylene glycol ricinoleate in a previous study [22] we decided to assess the effects on the growth performances, nutrient digestibility, hepatic fatty acid profile and excreta nitrogen ammonia content of glycerol polyethylene glycol ricinoleate and bi-distilled oleic acid in powder form supplemented in reduced-energy diets for broiler ROSS 308.

## 2. Materials and Methods

### 2.1. Ethical Approval

The present study was evaluated and approved by the Animal Welfare Committee of the University of Milan (protocol n°OPBA_88_2022, 9 September 2022).

### 2.2. Experimental Design, Diets and Animal Housing

A total of 720 male broiler ROSS 308 chicks (42.39 ± 0.65 g) were transferred to the experimental facilities of the University of Milan (Lodi via dell’Università 6, 26900, Italy) at 1 d of age from a commercial hatchery (Azienda Agricola Pollo Monteverde, 25035, Ospitaletto, Italy). Chicks were vaccinated for New Castle disease and infectious bronchitis at 1 d.

Immediately after arrival at the experimental facilities, chicks were weighed, and a randomization process was applied to allocate animals into 4 homogenous groups. Groups were assigned to guarantee body weight homogeneity. Chicks were housed in thirty-six pens, with twenty chicks per pen. Each experimental group was formed by 9 pens that were randomly allocated. Groups were constituted as follows: a positive control group fed a standard basal diet (PC), a negative control group fed a reduced-energy diet (NC, −70 kcal/kg of complete feed), a group fed the NC diet + 250 mg/kg of glycerol polyethylene glycol ricinoleate and bi-distilled oleic acid (EMUL1) and a group fed the NC diet + 500 mg/kg of glycerol polyethylene glycol ricinoleate and bi-distilled oleic acid (EMUL2). A 3-phase feeding program was applied; a starter (1–10 d), a grower (11–21 d) and a finisher phase (22–42 d) were considered. Crumbled feed was administered during the starter and grower phases, whereas pellet (2.5 mm) was administered in the finisher period. Feed was provided by a local feed mill (Agricom International, Pognano, 24040, Italy) and, to avoid cross contamination, PC and NC feeds were prepared prior to EMUL1 and EMUL2. Table 1 and Table 2 show diets and related chemical analyses, respectively. PC and NC diets differed for soybean oil content, which was reduced in the NC diet to reach a difference of 70 kcal/g of complete feed in terms of apparent metabolizable energy. The treatment was constituted by an emulsifier composed of 30% glycerol polyethylene glycol ricinoleate from ethoxylated castor oil (E484) and 30% bi-distilled oleic acid solidified through nebulization on silica particles (40%) to obtain a white powder form (Nutriemul P, Sevecom part of Barentz, Paderno Dugnano, 20037, Italy).

Each pen ensured a density within 33 kg/m^2^ as required by current legislation [27]. Pen floors were covered with wooden shavings. In each pen, two feed trays and 4 nipples for water distribution were provided; water and feed were available ad libitum. Environmental conditions were routinely checked and settled according to European Directive 2007/43/EC [27]. At the start of the trial, chicks were allocated with an air temperature corresponding to 32 °C, at chicks’ height. Temperature was gradually modulated to reach 22 °C on 28 d of the trial. Relative humidity was maintained at 65%. Temperature and humidity were monitored through automated sensors at chicks’ height. Ventilation was controlled automatically to maintain air quality. A total of 23 h of artificial light was guaranteed at the allocation of the chicks. Within the first seven days of the trial, artificial daylight was gradually regulated until reaching 16 h of light and 8 h of darkness as suggested by European Directive 2007/43/EC [27].

At the end of the trial (42 d), all the chickens were brought to a slaughterhouse placed 75 km away from the experimental facilities (Pollo Valcalepio, 24060, Telgate, Italy), following an overnight fast. One subject per replicate was selected based on the average weight of the pen to perform liver sampling.

### 2.3. Growth Performance

Body weight was evaluated at 1, 10, 21 and 42 d. Average daily gain (ADG), average daily feed intake (ADFI), feed conversion ratio (FCR) and feed efficiency (FE) were determined for the starter (1–10 d), grower (11–21 d), finisher (22–42 d) and the whole experimental period (1–42 d).

### 2.4. Feed and Excreta Analyses and Apparent Total Tract Nutrient Digestibility (ATTD)

The apparent total tract digestibility (ATTD) of nutrients was calculated considering acid-insoluble ashes (AIA) as undigestible markers. Siliceous earth (Celite, Merck KGaA, Darmstad, Germany) was administered at 0.5% in finisher feeds to increase AIA dietary content [28].

After three days of adaptation, at 24 and 42 d, pooled fresh excreta samples were collected directly from polyethylene trays placed over bedding following the methodology reported by Zampiga et al. [29]. Excreta samples (1.00 g) were dried at 70 °C for 24 h and ground at 1 mm screen prior to analysis. AIA in excreta and feed samples were assessed by boiling aliquots of samples in 4 N HCL for a total time of ten minutes and by filtering the resulting slurry through filter papers (Whatman N.541). Filters were washed with distilled water and then muffle-dried at 600 °C overnight.

Percent recovery was obtained using the following calculation:

AIA % = (Fx − Fy)/Fx × 100(1)

where Fx and Fy represent the initial and final filter weight, respectively. Gross energy in feed and excreta samples was determined through an adiabatic calorimetric bomb (IKA 4000, Staufen, Germany).

In addition, excreta and diets samples were analyzed for their crude protein (CP) content using the macro-Kjeldahl technique [30]. Ether extract was evaluated as described by Thiex et al. [31] through AOAC official method 2003.05. Total Ash content was established through AOAC 942.05 methodology [32]. The apparent total tract digestibility (ATTD) coefficient was based on the AIA content in the diet, considered a marker and calculated following the description given by Maharjan et al. [33] with the following equation:

ATTD Coefficient = 1 − [(Ax/Ay) × (Ny/Nx)](2)

where Ax represents AIA content in feeds and Ay the concentration of AIA in excreta. On the other hand, Nx stands for the nutrient content in diets, whereas Ny is the variable linked to the nutrient content in excreta samples.

Apparent metabolizable energy (AME) values were calculated following the indications depicted by Jimenez-Moya et al. [34] by multiplying the GE ATTD coefficient of excreta with the GE of the diet. Moreover, AME corrected per nitrogen (AMEn) content was calculated as suggested by Maharjan et al. [35]; the results are expressed in kcal/kg of complete feed. Nitrogen ammonia (NH_4_^+^-N) content in excreta was assessed through distillation [36] and results are expressed as mg/g of excreta on dry matter basis. For all the previous evaluations, biological triplicates of pooled samples were considered.

### 2.5. Lipid Extraction and Fatty Acid Characterization in Hepatic Tissue

Hepatic tissue lipids were extracted through the Folch extraction method, with minor modifications as described by Serini et al. [37]. Tissues were homogenized through a chloroform/methanol 1:2 solution and lipid extract was recovered through centrifugation. Subsequently, two extractions were performed through chloroform/methanol, 2:1 and 1:1 (*v*/*v*), respectively. A total of 0.045 mM of 3,5-di-tert-4-butylhydroxytoluene (BHT) was contained in solvents used for extraction to avoid PUFA oxidation.

The fatty acid composition was determined by gas chromatography (Shimadzu GC-2025, himadzu, Kyoto, Japan) as described by Ungaro et al. [38]. Fatty acid methyl esters (FAMEs) were obtained by lipid derivatization (sodium methoxide in methanol 3.33% (*w*/*v*)). C17:0 triglyceride was added to samples for correcting the reaction yield and recovery. Quantitative analysis was calibrated through a standard mixture (Sigma Aldrich, Milano, Italy) containing all fatty acid methyl esters. Biological triplicates were considered for hepatic lipid extraction and fatty acid characterization. Results were expressed as percentage of individual fatty acid methyl esters. Enzymatic activity related to hepatic fatty acids was assayed through specific FA ratios to estimate the activity of desaturase Δ5D (20:4n-6/20:3n-6), Δ6D (18:3n-6/18:2n-6), stearoyl-CoA desaturase 1 (SCD-1; 16:1n-7/16:0, SCD-16 and 18:1n-9/18:0) and elongases Elovl-5 (20:3n-6/18:3n-6) and Elovl-6 (18:0/16:0) as reported by Drag et al. [39].

### 2.6. Statistical Evaluations

Data referring to growth performance, feed consumption, nutrient digestibility, energy utilization (AME and AMEn), excreta nitrogen ammonia content and liver fatty acid profile were analyzed through a GLM procedure of SAS in a randomized block design (SAS programme Version 9.2, SAS Institute Inc., Cary, NC, USA).

Post hoc evaluation was assessed through a Tukey test to discriminate multiple contrast comparisons of mean values of the four groups. Data were considered statistically significant for *p* < 0.05 and highly significant for *p* < 0.01. All the data are presented as mean ± standard error mean (SEM). Pen represented the experimental unit for growth performances, nutrient digestibility, energy utilization and nitrogen ammonia content assessment. Single chickens were considered for hepatic fatty acid profiles evaluations.

## 3. Results

### 3.1. Growth Performance Evaluation

Performance evaluations are presented in Table 3. At 10 d, 500 mg/kg of solidified glycerol polyethylene glycol ricinoleate and bi-distilled oleic acid enhanced the body weight of EMUL2 chicks in comparison to PC (*p* < 0.05) and EMUL1 (*p* < 0.05). At 21 d, EMUL2 chickens showed higher BW in comparison to PC (*p* < 0.01) and NC (*p* < 0.01). Supplementing 250 mg/kg and 500 mg/kg of solidified glycerol polyethylene glycol ricinoleate and bi-distilled oleic acid enhanced the final BW of chickens in comparison to PC (*p* < 0.01) and NC (*p* < 0.01).

During the starter phase (0–10 d), EMUL2 chicks showed better average daily gain (ADG) in comparison to EMUL1 (*p* < 0.05) and to PC (*p* < 0.05). EMUL2 chickens demonstrated higher ADG in comparison to NC during the grower period (*p* < 0.01). Moreover, during the finisher phase, EMUL1 and EMUL2 chickens showed better ADG in comparison to PC (*p* < 0.01) and NC (*p* < 0.01). In addition, the 1–42 d period showed that 500 mg/kg of solidified glycerol polyethylene glycol ricinoleate and bi-distilled oleic acid enhanced the ADG of EMUL2 chickens in comparison to NC (*p* < 0.01) and PC (*p* < 0.01). During the finisher phase, average daily feed intake (ADFI) was increased in EMUL2 chickens in comparison to NC (*p* < 0.05). Considering the overall trial period, ADFI increased in both treatment groups in comparison to NC (*p* < 0.05). The feed conversion ratio (FCR) was higher in EMUL1 chickens compared to PC in the finisher phase (*p* < 0.05) and during the overall trial period (*p* < 0.05). Finally, no differences in mortality were detected during the trial among groups (*p* > 0.05).

### 3.2. Nutrients’ Apparent Total Tract Digestibility and Energy Utilization

The results referring to the nutrients’ apparent total tract digestibility (ATTD) and energy utilization are shown in Table 4. At 24 d, both dosages of solidified glycerol polyethylene glycol ricinoleate and bi-distilled oleic acid enhanced dry matter digestibility in comparison to PC (*p* < 0.01). On the other hand, dry matter digestibility increased in EMUL1 and EMUL2 groups in comparison to PC at 42 d (*p* < 0.05).

Nonetheless, at the end of the trial ash digestibility increased in EMUL1 chickens in comparison to PC (*p* < 0.05) and NC (*p* < 0.05).

Interestingly, at 24 d crude protein digestibility was conditioned by supplementing 500 mg/kg of solidified glycerol polyethylene glycol ricinoleate and bi-distilled oleic acid as EMUL2 chickens showed higher ATTD when compared to NC (*p* < 0.05). In addition, at 42 d EMUL2 chickens demonstrated increased crude protein digestibility in comparison to PC (*p* < 0.01) and NC (*p* < 0.01) chickens.

Both EMUL1 and EMUL2 showed higher ether extract digestibility at 24 d when compared to NC (*p* < 0.05). Furthermore, at the end of the trial EMUL2 showed enhanced ether extract digestibility when compared to PC (*p* < 0.01) and NC (*p* < 0.01).

EMUL2 evidenced higher gross energy (GE) digestibility at 24 d (*p* < 0.05). At 42 d, supplementation with 500 mg/kg of solidified glycerol polyethylene glycol ricinoleate and bi-distilled oleic acid enhanced GE digestibility in comparison to both NC (*p* < 0.01) and PC (*p* < 0.01). Moreover, 500 mg/kg of glycerol polyethylene glycol ricinoleate and bi-distilled oleic acid enhanced the apparent metabolizable energy availability of EMUL2 diets in comparison to NC (*p* < 0.05). Finally, at 42 d EMUL1 and EMUL2 showed higher energy values corrected for nitrogen content (AMEn) in comparison to NC (*p* < 0.05).

### 3.3. Nitrogen Ammonia Content in Excreta

**Figure 1:**
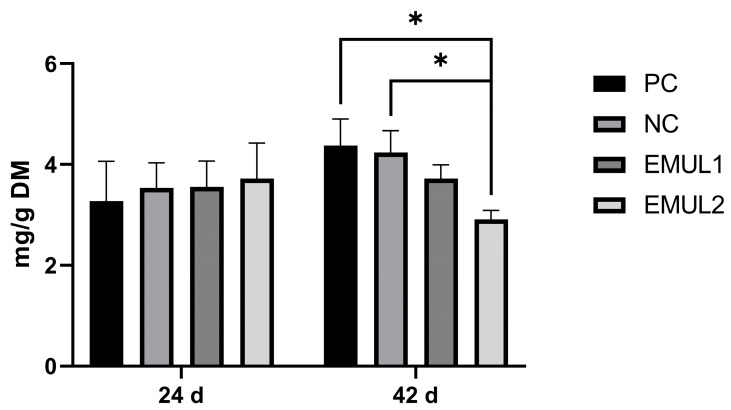
Nitrogen ammonia content in excreta evaluated at 24 and 42 d. Values are presented as means ± SEM. Pairwise comparisons mark statistically significant differences as follows: * = *p* < 0.05. Abbreviations = PC: positive control; NC: negative control; EMUL1: emulsifier supplemented at 250 mg/kg of complete feed; EMUL2: emulsifier supplemented at 500 mg/kg of complete feed.

Nitrogen ammonia (NH_4_^+^-N) content results are shown in Figure 1. No differences were detected among groups at 24 d (*p* > 0.05)

However, EMUL2 excreta highlighted a significant reduction in terms of NH_4_^+^-N content at 42 d when compared to PC (2.35 ± 0.73 mg/g vs. 4.82 ± 0.49 mg/g; *p* < 0.05). In addition, at 42 d EMUL2 excreta were characterized by a lower NH_4_^+^-N content in comparison to NC (2.35 ± 0.73 mg/g vs. 5.02 ± 0.72 mg/g; *p* < 0.01).

### 3.4. Hepatic Fatty Acid Profile and Deducted Enzymatic Activity

The fatty acid profile of liver samples is shown in Table 5. Fatty acid determination in hepatic tissue sampled at 42 d revealed a significantly lower stearic acid (C18:0) content in EMUL1 samples in comparison to NC (*p* < 0.05) and EMUL2 (*p* < 0.05).

**Figure 2:**
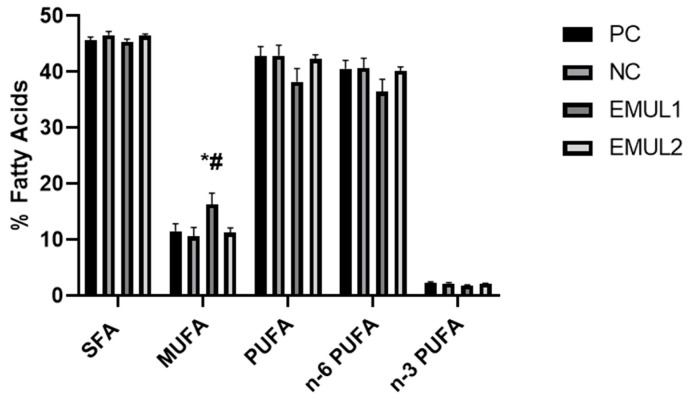
Relative values of fatty acid classes’ hepatic tissues shown at the end of the trial (42 d). Values are presented as means ± SEM. Pairwise comparisons mark statistically significant differences as follows: * = *p* < 0.05 vs. NC and # = *p* < 0.05 vs. EMUL2. Abbreviations = PC: positive control; NC: negative control; EMUL1: glycerol polyethylene glycol ricinoleate and bi-distilled oleic acid supplemented at 250 mg/kg of complete feed; EMUL2: glycerol polyethylene glycol ricinoleate and bi-distilled oleic acid supplemented at 500 mg/kg of complete feed; SFA: saturated fatty acid; MUFA: monounsaturated fatty acid; PUFA: polyunsaturated fatty acid; n-6 PUFA: n-6 or ω-6 polyunsaturated fatty acid; n-3 PUFA: n-3 or ω-3 polyunsaturated fatty acid.

**Figure 3:**
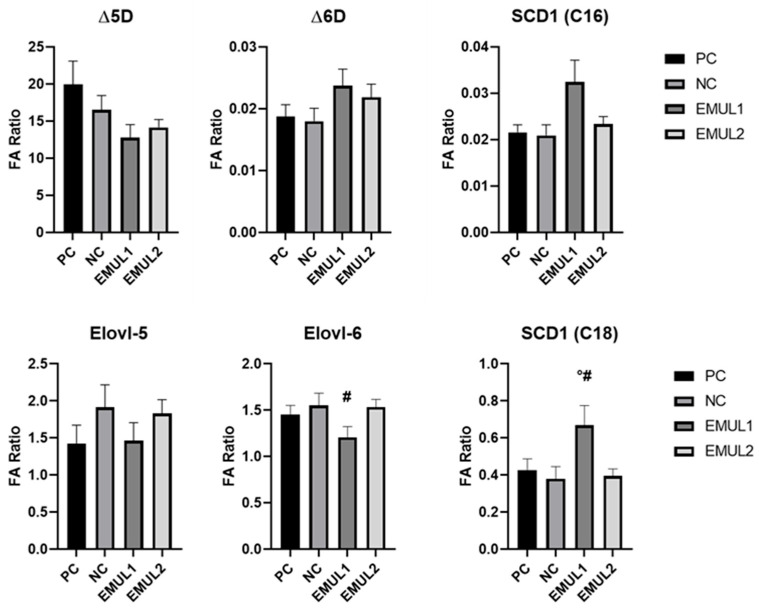
Indexes for desaturation and elongation enzymes involved in lipogenesis activity. Data are presented as mean ± SEM. Pairwise comparisons mark statistically significant differences as follows: # *p* < 0.05 vs. EMUL2 and ° *p* < 0.05 vs. PC. Abbreviations = PC: positive control; NC: negative control; EMUL1: glycerol polyethylene glycol ricinoleate and bi-distilled oleic acid supplemented at 250 mg/kg of complete feed; EMUL2: glycerol polyethylene glycol ricinoleate and bi-distilled oleic acid supplemented at 500 mg/kg of complete feed; Δ5D: delta-5-desaturase; Δ6D: delta-6-desaturase; SCD1: stearoyl-CoA desaturase 1; Elovl-5: elongase-5; Elovl-6: elongase-6.

On the other hand, oleic acid (C18:1) was significantly higher in EMUL1 when compared to NC (*p* < 0.05) and EMUL2 (*p* < 0.05). Moreover, PC highlighted enhanced levels of linoleic acid (C18:2) when compared to EMUL2 (*p* < 0.05). Interestingly, Dihomo-γ-linolenic acid (C20:3) was significantly higher in EMUL2 samples than in EMUL1 (*p* < 0.05) and PC (*p* < 0.05). In addition, arachidonic acid (C20:4) was higher in EMUL2 in comparison to EMUL1 (*p* < 0.05). The lipid distribution of the main fatty acid classes isolated in hepatic tissues collected at 42 d is shown in Figure 2. Overall, the monounsaturated fatty acid (MUFA) content was higher in EMUL1 samples in comparison to NC and EMUL2 (*p* < 0.05). Indexes for desaturation and elongation enzymes involved in lipogenesis activity were deducted and are shown in Figure 3. Interestingly, stearoyl-coenzyme desaturase 1 (SCD-1)-deducted activity was higher in EMUL1 samples when compared to that in PC and EMUL2 (*p* < 0.05). Elovl-6 activity was higher EMUL2 samples in comparison to EMUL1 (*p* < 0.05).

## 4. Discussion

As suggested by Kamran et al. (2020), vegetal oil reduction can be a valuable strategy to positively modulate broilers’ growth performance in the presence of emulsifiers’ dietary inclusion [3]. In the present study, glycerol polyethylene glycol ricinoleate and bi-distilled oleic acid in powder form positively modulated the growth performances of EMUL1 and EMUL2 animals.

At 10 d, the administration of 500 mg/kg of glycerol polyethylene glycol ricinoleate and bi-distilled oleic acid in powder form significantly enhanced the BW of chicks in a more effective way than 250 mg/kg and PC diets. These results can be explained by the presence of higher soybean oil inclusion in the PC diet, as young birds normally struggle to efficiently digest lipids, which may also explain the higher efficacy of EMUL2 treatment during the starter phase [40,41]. Furthermore, 500 mg/kg of glycerol polyethylene glycol ricinoleate and bi-distilled oleic acid in powder form enhanced the BW and the ADG of cockerel in comparison to NC at the end of the grower phase. These results are in accordance with a previous study which showed how the administration of 0.10% and 0.15% of lysophospholipid enhanced the BW and BWG of birds fed reduced-energy (−150 kcal/kg) diets [42].

The presented results showed that the positive and negative control groups were characterized by not-statistically-different growth performance during the trial. Oketch et al. (2022) underlined no detrimental effects on broiler Ross 308 performance when reducing the dietary metabolizable energy of 100 kcal/kg [43]. These observations are also in accordance with previous studies [44,45,46].

In the present study, EMUL2 chickens evidenced better growth performance related to a higher feed intake during the finisher phase. On the other hand, the EMUL1 group showed better feed conversion rates (FCRs) when considering the overall period of the trial. Emulsifiers can favor nutrient digestibility, bringing better conversion rates when modulating energy densities [47]. Interestingly, Kamran et al. (2020) demonstrated higher FCRs when considering the supplementation of 0.035% of glycerol polyethylene glycol ricinoleate in soybean oil-based diets [3]. In our previous study, the liquid form of glycerol polyethylene glycol ricinoleate and bi-distilled oleic acid was administered at 500 mg/kg during the finisher phase and enhanced the ADFI of broilers, leading to better final BWs and FCRs [48]. Therefore, our results are in line with previous findings for what concerns EMUL1 treatment. On the other hand, EMUL2 did not show improvements in FCR due to enhanced feed intake. In addition, no differences between EMUL1 and EMUL2 were detected in the final BWs. This result can be justified by a compensatory response to energy depletion supported by glycerol polyethylene glycol ricinoleate and bi-distilled oleic acid supplementation in EMUL2 [19].

To the best of our knowledge, only a recent study by Wealleans et al. [26] compared the effects of emulsifiers liquid and dry forms. In particular, the authors tested a combination of lysolecithin, a synthetic emulsifier and monoglycerides, finding better advantages in terms of growth performance when adding the liquid form. Nonetheless, differences in terms of product composition must be pointed out. Therefore, as consistent studies specifically focused on liquid and dry forms of glycerol polyethylene glycol ricinoleate and bi-distilled oleic acid are not available to date, it can be concluded that further research is needed to better assess the higher effectiveness of emulsifiers’ dry forms over liquid ones or vice versa. Overall, our results confirmed the positive effect of glycerol polyethylene glycol ricinoleate and bi-distilled oleic acid in powder form on chickens’ growth performance while reducing vegetal oil dietary inclusion, leading to lower feeding costs [49].

Exogenous emulsifiers positively shape nutrient digestibility, as indicated by previous studies [1,2,3,4]. Dry matter digestibility was increased at 24 d and 42 d by both emulsifier dosages. According to Gholami et al. (2024), the dietary supplementation of 1000 and 1500 mg/kg of a lysophopholipid mixture enabled an increase in dry matter digestibility when considering 100 kcal/kg AME reduction [50]. In addition, Kamran et al. (2020) showed better dry matter digestibility when supplementing glycerol polyethylene glycol ricinoleate in soybean oil-based diets [3]. Our results confirmed what the author reported, showed positive effects in terms of DM digestibility when supplementing glycerol polyethylene glycol ricinoleate and bi-distilled oleic acid in powder form in reduced-energy diets.

Interestingly, glycerol polyethylene glycol ricinoleate and bi-distilled oleic acid in powder form supplementation increased ash digestibility in EMUL1 and EMUL2 broilers. A previous study by Dierick and Decuypere (2004) showed that the capacity of a combination of microbial derived lipase (0.05%) and an emulsifier (0.3%) enhanced the ATTD of ash in growing pigs [51]. In our study, better ash digestibility was achieved by supplementing glycerol polyethylene glycol ricinoleate and bi-distilled oleic acid in powder form. However, the available research lacks consistency when considering the effects of emulsifiers on ash digestibility. Therefore, further research is needed to assess the effects of emulsifiers on ash digestibility.

Furthermore, emulsifiers can influence protein digestion. Ahmadi-Sefat et al. (2022) found a linear increase in crude protein’s apparent ileal digestibility when administering a mixture of lysophospholipids in broiler chickens’ diets [22]. In a previous study conducted by Boontiam et al. (2019), there was a linear increase in the CP digestibility of growing broilers fed low-energy diets (−150 kcal/kg) supplemented with lysophospholipids [42]. Thus, our results are in line with these findings as the dietary inclusion of 500 mg/kg of glycerol polyethylene glycol ricinoleate and bi-distilled oleic acid in powder form enhanced crude protein digestibility in both treated groups.

In addition, emulsifiers can favor lipid digestibility by enhancing pancreatic lipase activity and promoting their absorption [52,53]. Roy et al. (2010) reported that supplementing broiler diets with glycerol polyethylene glycol ricinoleate increased lipids’ digestibility [54]. Zhao et al. (2015) found higher lipid digestibility supplementing lysophospholipids in weanling piglets diets [55]. Kamran et al. (2020) demonstrated that supplementing glycerol polyethylene glycol ricinoleate can positively influence ether extract digestibility in broiler diets [3]. Jalal et al. (2024) showed that emulsifier supplementation combined with dietary energy modulation can lead to improvements in ether extracts and crude protein digestibility [47]. Exogenous emulsifiers can enhance the active surface of lipids in feed particles, leading to a more pronounced activity of digestive enzymes that further modulates proteins’ digestibility [56,57]. Thus, emulsifiers may increase the digestive enzymatic activity towards dietary proteins components by modulating lipids digestibility [58,59]. Indeed, emulsifiers can act on bile acids and phospholipids, which have a central role in conditioning the digestion kinetics of proteins and amino acids throughout the stomach and intestine [59]. Nonetheless, further evaluations concerning the effects of glycerol polyethylene glycol ricinoleate and bi-distilled oleic acid in powder form on digestive enzymes’ activity are needed to confirm this mode of action.

In the present study, supplementing glycerol polyethylene glycol ricinoleate and bi-distilled oleic acid in powder form enhanced the apparent metabolizable energy of finisher diets. Our results agree with Tan et al. (2016), who reported increased AME at the end of the 5th week of life of Cobb 500 broilers administered a premix containing polyethylene glycol ricinoleate [60]. According to Oliveira et al. (2023), emulsifiers’ supplementation in diets characterized by energy depletion due to reduced soybean oil inclusion can positively influence dietary energy availability [61].

Taken together, the discussed results confirmed that glycerol polyethylene glycol ricinoleate and bi-distilled oleic acid in powder form supplementation ameliorated nutrients’ digestibility, leading to advanced dietary energy availability.

Due to microbial fermentation, gaseous ammonia release in the atmosphere forms small particles of particular matter (PM10; <10 µm) which are included among the most environmentally impactful air pollutants [62]. In our study, EMUL2 groups registered lower NH_4_^+^-N levels in excreta at 42 d. Interestingly, the same group had better CP digestibility at the end of the trial. Optimizing dietary protein utilization may lead to reduced nitrogen losses and ammonia emissions [63]. Nonetheless, the available literature lacks clear correlations among dietary emulsifiers inclusion, crude protein digestibility and excreta nitrogen ammonia concentrations. Therefore, further research is needed to evaluate the effects of glycerol polyethylene glycol ricinoleate and bi-distilled oleic acid in powder form on nitrogen balance and ammonia emissions.

Lipid synthesis in the liver is the main metabolic source of fats in chickens [64]. Vegetal oils are useful for guaranteeing optimal fatty acid uptake, which is helpful in supporting important biological functions such as fat-soluble vitamins’ transportation and utilization [34]. In liver, lipogenesis is catalyzed by a series of linked enzymes, such as acetyl-CoA carboxylase (ACC), fatty acid synthase (FAS) and stearoyl CoA desaturase 1 (SCD-1) [65]. SCD-1 is an endoplasmic enzyme and promotes the biosynthesis of monounsaturated fatty acids (MUFAs) from dietary or de novo synthesized saturated fatty acids [65]. Interestingly, the EMUL1 treatment group displayed the highest hepatic oleic acid (C18:1) content when compared to NC and EMUL2, whereas the opposite trend was depicted when analyzing the stearic acid (C18:0) content. These results can be linked to a the higher stearoyl-coenzyme A desaturase 1 (SCD1) activity. Indeed, SCD1 has a primary role in modulating stearate (C18:0) desaturation in chickens [65]. On the other hand, higher levels of oleic acid (C18:1) markedly conditioned the total MUFA content in the EMUL1 treatment group [66]. Furthermore, the increased presence of linoleic acid (C18:2) in PC group livers can be justified by the higher soybean oil dietary content, confirming the capacity of vegetal oils to increase essential PUFA contents [67].

On the other hand, the EMUL2 group revealed higher arachidonic (C20:4) and dihomo-γ-linolenic acid (C20:3) variations in the liver. Elongase of very long-chain fatty acids (*ELOVL*) are a family of pivotal enzymes which regulate the formation of long-chain monounsaturated and saturated fatty acid and lipid deposition in tissues [68,69]. In addition, *ELOVL-6* activity was found to be lower in EMUL1 samples compared with EMUL2 samples. Higher levels of stearic acid (C18:0) were detected in EMUL2 samples at the end of the trial. Therefore, it can be speculated that stearate levels might be influenced by the elongation of pre-existing palmitate (C16:0) due to enhanced *ELOVL-6* activity [70]. These changes may be further related to chicken meat quality variations, as fat deposition is regulated by *ELOVL* genes and influences different quality attributes [71]. The collected results may highlight the capacity of glycerol polyethylene glycol ricinoleate and bi-distilled oleic acid in powder form’s inclusion in higher dosages to modulate processes of fatty acids enzymatic elongation. Nonetheless, further evaluations should be useful in clarifying the potential of glycerol polyethylene glycol ricinoleate and bi-distilled oleic acid in targeting the enzymatic activity of the liver and influencing chicken meat quality.

## 5. Conclusions

Supplementation with an emulsifier composed of glycerol polyethylene glycol ricinoleate and bi-distilled oleic acid in powder form in broiler diets with reduced energy content improved growth performance, nutrient digestibility and liver fatty acid composition while reducing nitrogen ammonia excretion. These findings highlight that the tested emulsifier can be a cost-effective strategy for sustainable broiler production considering large-scale use.

## Figures and Tables

**Table 1:** Experimental diets administered to animals during the trial.

	Starter		Grower		Finisher	
Ingredients, % as Fed	PC	NC, EMUL1, EMUL2	PC	NC, EMUL1, EMUL2	PC	NC, EMUL1, EMUL2
Maize meal	52.17	49.00	47.51	44.51	53.28	53.28
Soybean meal (46% CP)	37.50	38.00	34.00	33.00	31.30	32.00
Wheat	2.00	5.30	10.00	15.20	5.00	5.50
Soybean oil	4.00	2.97	5.00	3.80	6.20	5.00
Sodium chloride	0.40	0.40	0.35	0.35	0.25	0.25
Calcium carbonate	1.20	1.20	1.00	1.00	0.95	0.95
Dicalcium phosphate	1.50	1.50	1.00	1.00	1.00	1.00
DL-methionine	0.34	0.34	0.30	0.30	0.25	0.25
L-threonine	0.11	0.11	0.09	0.09	0.04	0.04
L-lysine HCL	0.28	0.25	0.25	0.25	0.23	0.23
Vitamins + trace elements ^1^	0.50	0.50	0.50	0.50	0.50	0.50
Celite	-	-	-	-	0.50	0.50
Chemical components, % as fed (calculated)						
ME, kcal/kg	3050	2980	3100	3030	3200	3130
Crude protein, %	21.80	21.80	20.20	20.20	19.40	19.40
Ether extract, %	6.30	5.30	6.20	5.00	8.00	7.20
Lysine, %	1.33	1.33	1.20	1.20	1.13	1.13
Calcium, %	0.90	0.90	0.75	0.75	0.70	0.70
Available phosphorus, %	0.60	0.60	0.55	0.55	0.50	0.50

^1^ Provided the following per kg of diet: vitamin A, 11,250 IU; vitamin D3, 5000 IU; vitamin E, 60 mg; MnSO_4_·1H_2_O, 308 mg; ZnSO_4_·1H_2_O, 246 mg; FeSO_4_·1H_2_O, 136 mg; CuSO_4_·5H_2_O, 39 mg; KI, 2.4 mg; Na_2_SeO_3_, 657 μg; 6-Phytase EC 3.1.3.26, 750 FTU; Endo-1, 4-beta-xylanase EC 3.2.1.8, 2250 U. Abbreviations: ME: metabolizable energy; PC: positive control; NC: negative control; EMUL1: 250 mg/kg of glycerol polyethylene glycol ricinoleate and bi-distilled oleic acid; EMUL2: 500 mg/kg of glycerol polyethylene glycol ricinoleate and bi-distilled oleic acid.

**Table 2:** Chemical analyses of experimental diets (*n* = 5 samples from each phase). Results of analyses are represented as mean ± standard deviations.

Chemical Components, % as Fed ^1^	Starter				Grower				Finisher			
	PC	NC	EMUL1	EMUL2	PC	NC	EMUL1	EMUL2	PC	NC	EMUL1	EMUL2
DM	89.34 ± 3.81	87.59 ± 2.97	89.23 ± 2.88	88.40 ± 2.71	87.62 ± 3.22	88.37 ± 3.30	88.12 ± 3.44	89.61 ± 3.27	89.17 ± 3.71	88.56 ± 3.49	89.04 ± 3.44	87.92 ± 3.31
CP	22.20 ± 0.71	22.00 ± 0.63	22.43 ± 68	22.67 ± 0.65	20.58 ± 0.78	20.32 ± 0.73	20.12 ± 0.70	20.27 ± 0.76	19.58 ± 0.62	19.81 ± 0.64	19.29 ± 0.67	19.77 ± 0.74
EE	6.00 ± 0.35	4.93 ± 0.58	4.90 ± 0.55	4.84 ± 0.59	6.88 ± 0.37	5.12 ± 0.31	5.19 ± 0.42	5.24 ± 0.39	7.89 ± 0.36	6.90 ± 0.33	6.85 ± 0.41	6.82 ± 0.40
Ash	4.00 ± 0.20	3.98 ± 0.23	3.80 ± 0.28	4.08 ± 0.26	4.12 ± 0.21	4.19 ± 0.25	3.91 ± 0.34	3.78 ± 0.31	5.41 ± 0.35	5.34 ± 0.29	5.62 ± 0.32	5.79 ± 0.33
GE, kcal/kg	4038 ± 54	3964 ± 51	3957 ± 56	3961 ± 53	4126 ± 52	4040 ± 57	4059 ± 54	4063 ± 50	4233 ± 52	4129 ± 55	4127 ± 49	4124 ± 58

^1^ Abbreviations = PC: positive control; NC: negative control; EMUL1: 250 mg/kg of glycerol polyethylene glycol ricinoleate and bi-distilled oleic acid; EMUL2: 500 mg/kg of glycerol polyethylene glycol ricinoleate and bi-distilled oleic acid; DM: dry matter; CP: crude protein; EE: ether extract; GE: gross energy.

**Table 3:** Performance parameters registered during the trial. All the values are intended as mean ± SEM. Different letters mark statistically significant differences shown after contrast comparisons (A,B = *p* < 0.01; a,b = *p* < 0.05).

Parameters	PC	NC	EMUL1	EMUL2	SEM	*p*-Value
BW (g)						
1 d	42.42	42.48	42.52	42.14	0.22	0.87
10 d	261.14 ^b^	266.83 ^ab^	260.49 ^b^	274.62 ^a^	3.16	<0.05
21 d	840 ^B^	828 ^B^	870 ^AB^	898 ^A^	13	<0.01
42 d	2609 ^B^	2558 ^B^	2846 ^A^	2774 ^A^	30	<0.01
ADG (g/d)						
1–10 d	23.74 ^b^	24.26 ^ab^	23.68 ^b^	24.96 ^a^	0.29	<0.05
11–21 d	57.92 ^AB^	56.11 ^B^	60.64 ^AB^	62.08 ^A^	1.26	<0.01
22–42 d	83.76 ^B^	82.12 ^B^	92.58 ^A^	89.07 ^A^	1.28	<0.01
1–42 d	62.60 ^B^	61.34 ^B^	68.36 ^A^	66.63 ^A^	0.74	<0.01
ADFI (g/d)						
1–10 d	26.73	26.56	27.01	27.14	0.32	0.91
11–21 d	79.06	75.96	77.91	79.35	1.18	0.89
22–42 d	151.11 ^ab^	144.32 ^b^	153.35 ^ab^	154.32 ^a^	2.54	<0.05
1–42 d	103.85 ^ab^	99.57 ^b^	104.80 ^a^	105.68 ^a^	1.33	<0.05
FCR						
1–10 d	1.12	1.09	1.14	1.08	0.01	0.82
11–21 d	1.37	1.35	1.29	1.28	0.03	0.75
22–42 d	1.80 ^b^	1.76 ^ab^	1.65 ^a^	1.73 ^ab^	0.03	<0.05
1–42 d	1.66 ^B^	1.62 ^B^	1.53 ^A^	1.59 ^AB^	0.02	<0.01
Mortality%						
1–42 d	2.22	1.66	2.22	1.66	0.01	0.68

Abbreviations = PC: positive control; NC: negative control; EMUL1: glycerol polyethylene glycol ricinoleate and bi-distilled oleic acid supplemented at 250 mg/kg of complete feed; EMUL2: glycerol polyethylene glycol ricinoleate and bi-distilled oleic acid supplemented at 500 mg/kg of complete feed; BW: body weight; ADG: average daily gain; ADFI: average daily feed intake; FCR: feed conversion ratio.

**Table 4:** Apparent total tract nutrient digestibility and energy utilization data analyzed at 24 and 42 d. All the values are reported as mean ± SEM. Different letters mark statistically significant differences shown after contrast comparisons (A,B = *p* < 0.01; a,b = *p* < 0.05).

Parameters	PC	NC	EMUL1	EMUL2	SEM	*p*-Value
DM						
24 d	0.934 ^b^	0.941 ^ab^	0.954 ^a^	0.953 ^a^	0.004	<0.05
42 d	0.941 ^B^	0.943 ^AB^	0.956 ^A^	0.961 ^A^	0.004	<0.01
Ash						
24 d	0.431	0.497	0.523	0.477	0.020	0.62
42 d	0.508 ^B^	0.534 ^B^	0.624 ^A^	0.556 ^AB^	0.023	<0.01
CP						
24 d	0.760 ^ab^	0.794 ^b^	0.811 ^ab^	0.832 ^a^	0.013	<0.05
42 d	0.723 ^B^	0.755 ^B^	0.774 ^AB^	0.803 ^A^	0.011	<0.01
EE						
24 d	0.936 ^ab^	0.917 ^b^	0.952 ^a^	0.950 ^a^	0.009	<0.05
42 d	0.877 ^B^	0.889 ^B^	0.924 ^AB^	0.936 ^A^	0.015	<0.01
GE						
24 d	0.746 ^b^	0.789 ^ab^	0.781 ^ab^	0.807 ^a^	0.013	<0.05
42 d	0.769 ^B^	0.779 ^B^	0.814 ^AB^	0.827 ^A^	0.011	<0.01
AME (kcal/kg)						
24 d	3094	3166	3139	3237	53	0.54
42 d	3190 ^ab^	3130 ^b^	3274 ^ab^	3318 ^a^	46	<0.05
AMEn (kcal/kg)						
24 d	3088	3140	3116	3212	59	0.46
42 d	3174 ^ab^	3099 ^b^	3241 ^a^	3285 ^a^	45	<0.05

Abbreviations = PC: positive control; NC: negative control; EMUL1: glycerol polyethylene glycol ricinoleate and bi-distilled oleic acid supplemented at 250 mg/kg of complete feed; EMUL2: glycerol polyethylene glycol ricinoleate and bi-distilled oleic acid supplemented at 500 mg/kg of complete feed; DM: dry matter; CP: crude protein; EE: ether extract; GE: gross energy; AME: apparent metabolizable energy; AMEn: apparent metabolizable energy corrected for nitrogen content.

**Table 5:** Lipidic composition (% of fatty acids) of the hepatic tissue collected at the end of the trial (42 d). All the values are reported as mean ± SEM. Different letters mark statistically significant differences (a,b = *p* < 0.05).

Parameters	PC	NC	EMUL1	EMUL2	SEM	*p*-Value
C16:0 (Palmitic)	18.90	18.60	21.02	18.46	0.60	0.08
C16:1 (Palmitoleic)	0.42	0.41	0.72	0.44	0.08	0.07
C18:0 (Stearic)	26.75 ^ab^	27.97 ^a^	24.39 ^b^	27.98 ^a^	0.84	<0.05
C18:1 (Oleic)	11.11 ^ab^	10.15 ^b^	15.63 ^a^	10.81 ^b^	1.25	<0.05
C18:2 (Linoleic)	27.59 ^a^	26.38 ^ab^	25.48 ^ab^	25.59 ^b^	0.49	<0.05
C18:3γ (γ-linolenic)	0.52	0.48	0.60	0.57	0.03	0.67
C18:3α (α-linolenic)	0.93	0.85	0.79	0.78	0.04	0.90
C20:3 (Dihomo-γ-linolenic)	0.64 ^b^	0.81 ^ab^	0.76 ^b^	0.94 ^a^	0.04	<0.05
C20:4 (Arachidonic)	11.81 ^ab^	12.99 ^ab^	9.59 ^b^	13.08 ^a^	0.81	<0.05
C20:5 (Eicosapentaenoic)	0.08	0.09	0.08	0.09	0.003	0.22
C22:5 (Docosapentaenoic)	0.41	0.45	0.35	0.46	0.02	0.15
C22:6 (Docosahexaenoic)	0.85	0.82	0.59	0.82	0.06	0.11

Abbreviations: PC: positive control; NC: negative control; EMUL1: glycerol polyethylene glycol ricinoleate and bi-distilled oleic acid supplemented at 250 mg/kg of complete feed; EMUL2: glycerol polyethylene glycol ricinoleate and bi-distilled oleic acid supplemented at 500 mg/kg of complete feed.

## Data Availability

Data are available upon reasonable request from the corresponding author.
